# The effect of impacted third molars on second molar external root resorption, a cross-sectional cone beam computed tomography study

**DOI:** 10.4317/medoral.25860

**Published:** 2023-10-12

**Authors:** Gökhan Gürses, Ali Akçakaya, Ahmet Aktı, Olgun Aydin

**Affiliations:** 1 Selcuk University, Dentistry Faculty, Department of Oral and Maxillofacial Surgery, Konya, Turkey; 2 Private Practitioner, Konya, Turkey; 3 Gdansk University of Technology, Department of Statistics, Gdansk, Poland

## Abstract

**Background:**

Third molars have the highest prevalence of impaction in teeth and can cause pathological damage on the adjacent second molars. This study aims to evaluate the effects of factors related to impacted third molars on external root resorption (ERR) in adjacent second molars using cone-beam computed tomography (CBCT).

**Material and Methods:**

In CBCTs, the effect of impacted third molars on the root surface of adjacent second molars was investigated. Inclusion criteria for subjects were being older than 16 and younger than 55, presence of at least one impacted third molar and adjacent second molar. Exclusion criteria were pathology, a follicle gap greater than 5 mm, crowned second molar, severe decay, an artifact on a radiologic image, and previous surgery on the second or third molars. The investigations were made based on age range, gender, tooth inclination, Pell-Gregory classification, retention type, contact area, root formation, pericoronal width, and tooth absence on the same quadrant for potential risk factors. The collected data were statistically analyzed with R software. The Chi-Square test was used to find out any significant difference. Logistic regression analyses were done for potential risk factors for ERR.

**Results:**

A total of 437 impacted third molars and adjacent second molars were investigated using CBCT. Of these, 381 met the inclusion criteria. Mesioangular and horizontal inclination, Pell-Gregory Class B-C, contact area, and retention type were found the statistically potential risk factors for ERR.

**Conclusions:**

The impacted third molar with horizontal or mesioangular position, and osseous retention, with Pell and Gregory Class B and C, are more likely to cause external root resorption in adjacent second molars.

** Key words:**Cone-beam CT, external root resorption, impacted third molar.

## Introduction

Etiologic factors in third molar impactions are dental crowding, malposition of permanent tooth germs, presence of supernumerary teeth, odontogenic tumors, abnormal eruption tract, thick fibrous mucosa, or overlying bone density (1). There is an ongoing discussion among surgeons on when to extract or follow up on impacted third molars, but the prophylactic extraction of an asymptomatic third molar is not an easy decision (2).

The impacted teeth often cause pathological conditions such as pericoronitis, odontogenic cysts and tumors, bone loss, and root resorption in adjacent teeth, leading to impaired oral function and discomfort (3). External root resorption (ERR) of second molars is a clinical condition that cannot be easily diagnosed. In two-dimensional radiographic studies, the prevalence of ERR in second molars has been reported as 0.3-7% (4,5). The diagnostic value of panoramic and periapical radiographs for identifying ERR is relatively low due to image distortion and their projective geometry. Cone-beam computed tomography (CBCT) detects 4.3 times more ERR than panoramic radiographs and has less radiation exposure than conventional computed tomography (6). With CBCT, clinical practitioners can investigate pathological conditions and adjacent anatomical structures related to impacted teeth in more detail.

In one study examining the impaction rate of third molar teeth, a rate of 54.1% was found (7). In another study, the frequency of impaction of the maxillary third molar was 43.2%, and the frequency of impaction of the mandibular third molar was 56.8% (8). In populations with a high prevalence of third molar impaction, risk factors for ERR should be identified, and preventive measures should be implemented.

ERR diagnosis is challenging early in the resorption because of a lack of symptoms. There is no doubt that the resorption will be advanced when diagnosed. After diagnosis, it may require root canal treatment, root resection, or tooth extraction. It would not be practical to obtain CBCT from all patients to diagnose ERR before it progresses. Instead, defining ERR risk factors and prophylactic extraction in patients with risk factors may be a vital step in ERR-induced second molar loss. In other words, determining the ERR risk factors would increase prophylactic impacted third molar extractions to avoid ERR of second molars.

Our study aims to examine the risk factors associated with impacted third molars that cause ERR via CBCT. In this way, we aim to prevent ERR-induced second molar tooth extractions by giving clinicians an idea of ERR when deciding on prophylactic extraction or following an impacted tooth. The null hypothesis is that missing teeth in the same quadrant, mesioangular and horizontal inclination, retention type, and the Pell and Gregory Class C are the potential risk factors for ERR on adjacent second molars.

## Material and Methods

A cross-sectional study was designed for making examinations and measurements on CBCT. The study was carried out with the approval of Selçuk University, Faculty of Dentistry Research Ethics Committee (2022/13).

CBCTs were taken between January 2019 and January 2020, for other diagnostic purposes, such as the surgical removal of third molars, the presence of pathology, orthodontic treatment, and implant planning. All CBCTs were obtained from the same device (Instrumentarium Dental, PaloDEx Group Oy Nahkelantie 160 FI-04300 TUUSULA, Finland). DVT images were obtained with 832.32 mGy x cm2 using an 8 cm x 15 cm FOV area, 0.250 mm voxel size, 90 kV, 5.0 mA, and an exposure time of 8.14.

Inclusion criteria were the presence of at least one impacted third molar and adjacent second molar, age range from 16 to 55, and adequate image quality for radiographic examination. Exclusion criteria were pathology, a follicle gap greater than 5 mm, crowned second molar, severe decay or artifact on a radiologic image, and previous surgery on the second or third molars.

Our primary variable was ERR. Possible risk factors were age, gender, impacted tooth inclination, retention type, pericoronal follicle width, contact area, missing tooth in the same quadrant, and root formation of the impacted tooth. Two specialists interpreted the CBCT scans in the axial, coronal, and sagittal planes.

Ericson and Kurol criteria were used to determine the presence and level of ERR on the root surface of the second molar. The classification was graded as follows: [1] no resorption, with an intact root surface (the cementum layer may have been lost), [2] slight resorption, with resorption up to half of the dentine thickness, [3] moderate resorption, with resorption of the dentine midway to reaching the pulp or further, with the pulp lining unbroken, and [4] severe resorption, with resorption reaching the pulp (9). When ERR was detected, it was categorized according to its location as cervical, middle, or apical root third.

The impaction type of mandibular third molar teeth was determined using the Pell and Gregory classification (10). Class A: the highest point of the third molar is at the same or higher level with the occlusal plane; Class B: the highest point of the third molar is between the occlusal plane and the cervical line of the mandibular second molar; Class C: the highest point of the third molar is below the cervical line of the mandibular second molar. The inclination of the third molars was determined using the Winter classification (11). The Winter classification was used to group the impacted third molars according to their apicocoronal axis. In vertical impaction, the second molar's long axis is parallel to the third molars' (-10°/+10°); in mesioangular impaction, the third molar is tipped in mesial direction (11°/79°); in distoangular impaction, the third molar is tipped to the distal direction (-11°/-79°); horizontal impaction, the third molar is a horizontal position (80°/100°) and the other impaction types (101°/-80°)(12). We excluded the other impaction types from the study. The retention status of the impacted third molar was determined as osseous or mucosal.

The contact areas were also recorded. The root formation development of the third molar was determined as follows: root tip formed closed, root tip formed open, 2/3 formed, and less than 2/3 formed. The follicle size of the impacted third molar was classified as follows: narrow (smaller than 1 mm), medium (between 1 to ≤3 mm), and wide (between 3 to ≤5 mm). Follicle sizes larger than 5 mm were excluded from the study.

The sample size was calculated as 380 with an effect size of 0.19, a significance level of 0.05, and a power of 0.8, and degrees of freedom were chosen as 3 since the highest number of categories used in the study is 4, and for ERR, there are two categories; in this case, degrees of freedom were calculated as (2-1) x (4-1) = 3 (13).

The Chi-Square test was used as an independence test to check whether the categorical variables were independent from each other in other words, to check whether there is any relationship between categorical variables. Logistic regression analysis examined the relationship between the potential risk factors and ERR in second molars. Cohen’s Kappa test evaluated inter-examiner agreement. The kappa coefficient was elucidated according to Landis and Koch (14). The statistical significance level (alpha) was determined as 0.05. R Programming language (version 4.0.5) was used for statistical analysis.

## Results

**Figure 1:**
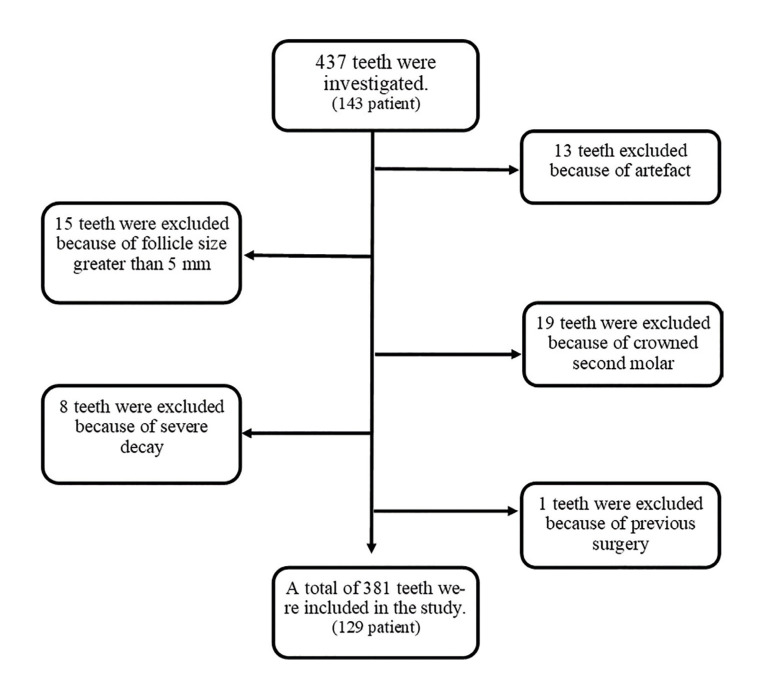
Flowchart of case selection.

A total of 437 impacted third molars and adjacent second molars were investigated with the CBCT volumes. Only 381 of them, belonging to 129 patients (63 males, 66 females), met the inclusion criteria (Fig. 1). The mean age is 24,7 (range: 16-55).

**Figure 2:**
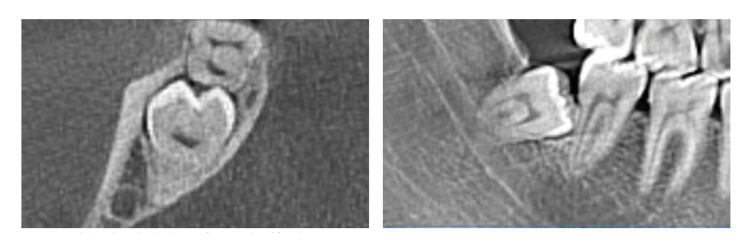
Axial and sagittal views of ERR Classification 2.

**Figure 3:**
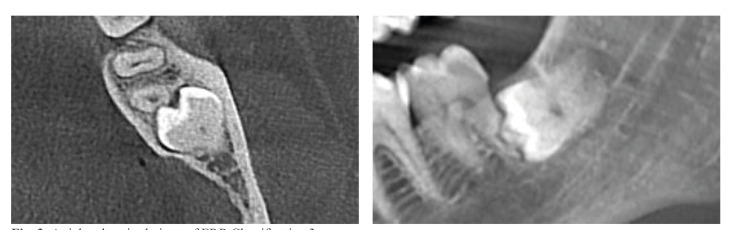
Axial and sagittal views of ERR Classification 3.

**Figure 4:**
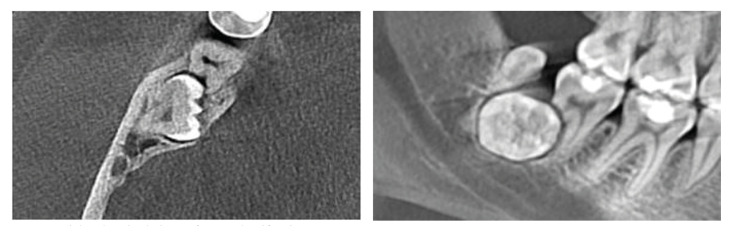
Axial and sagittal views of ERR Classification 4.

174 cases did not have ERR/Class 1. The common prevalence of ERR in our study population was 54,3% (207 cases). Based on ERR classification, percentages were 37,7% (144 cases)/Class 2 (Fig. 2); 12,8% (49 cases)/Class 3 (Fig. 3); 3,6% (14 cases)/Class 4 (Fig. 4). Age ranges could not be evaluated because group numbers (338/48) did not fulfill the chi-square test assumptions. Gender and jaws did not affect ERR (*p*=0,292, *p*=0,650; respectively). There was a statistically significant correlation between the inclination of impacted third molars and the presence of ERR. Mesioangular and horizontal positions were more prone to create ERR (*p*=0.003). The presence of ERR changed significantly according to the Pell and Gregory Class B and C (*p*=0.000).

A significant difference was found between the two retention types. The incidence of ERR was higher in osseous retention cases (*p*=0.007). Based on the contact area, the ERR occurrence in the cervical third was significantly different (*p*=0.006). The root formation did not create any difference (*p*=0.081). Similarly, the relationship between pericoronal follicle width, missing teeth in the same quadrant, and ERR was examined, and no significant difference was found (*p*=0.063, *p*=0.151, respectively). All group prevalences of ERR are indicated in Table 1.

Cohen’s Kappa test showed the inter-examiner agreement was found to be almost perfect (0.82). Logistic regression was applied to the dataset, backward elimination technique was used. After backward elimination, the final logistic regression contained jaws, distoangular inclination, vertical inclination, Pell-Gregory Classification (Class B), and Pell-Gregory Classification (Class C) variables. Logistic regression analysis revealed that gender did not have an effect on ERR occurrence. Distoangular inclination, vertical inclination, and Pell-Gregory Classification (Class C) had an effect on the occurrence of ERR. The result of the multivariate analysis is combined in Table 2.

**Table 1:**
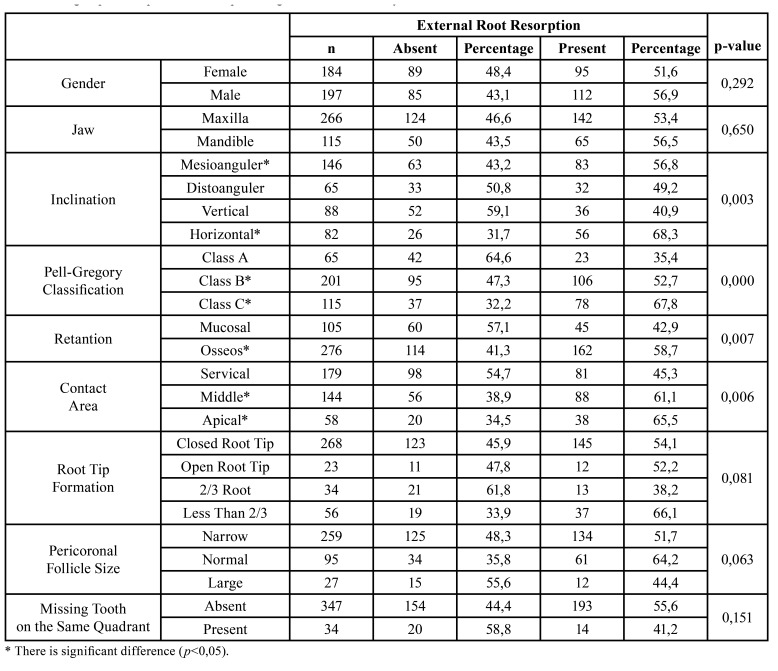
All groups’ ERR prevalance and percentages and bivariate analysis results.

**Table 2:**
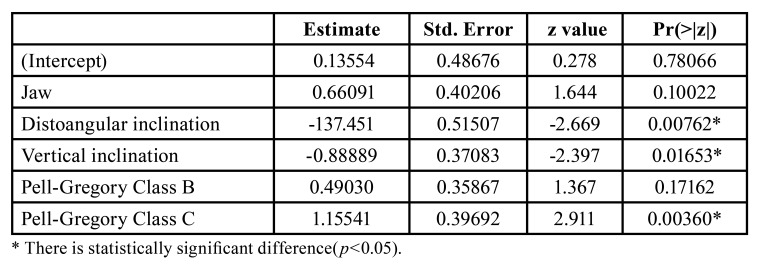
Multivariate analysis results.

## Discussion

Impacted teeth can cause many pathological conditions such as pericoronitis, bone loss, infection, dental caries, cheek injuries, odontogenic cysts or tumors, and ERR in adjacent second molars (6). ERR on the second molar caused by the impacted third molar is challenging in clinical diagnosis and estimation. Establishing a treatment plan when ERR is suspected presents difficulties for clinicians (15). It is difficult to diagnose early in the resorption process, so it may require invasive treatment. Extraction of impacted third molars removes mechanical stress and risks in second molars and prevents the inflammatory process and possible resorption. In the case of bacterial invasion into the pulp due to severe resorption, endodontic treatment is required. Our study aimed to investigate the predictive risk factors for ERR. In this direction, we revealed risk factors of ERR that are mesioangular and horizontal inclination, Pell-Gregory Class B and C, osseous retention, and middle and apical contact areas.

With CBCT, the position of the mandibular third molar, its relationship with the second molar, and related pathologies are evaluated tridimensionally in more detail. CBCT studies that examine third and second molars show a much higher incidence of ERR in comparison to studies focusing on panoramic or periapical radiography (6,16-18). In addition, 2D methods can cause misinterpretation or uninterpretability of images due to overlapping and distortion (19). Researchers who previously investigated second molar ERR on CBCT reported a prevalence of 22.8% (6) and 21% (20). We found a prevalence of 54,3%. There may be many parameters that play a role in the difference. Population selection may be the foremost. But at the same time, the low number of mesioangular and horizontal impacted third molars (6) or a single observer (20) may also have led to such differences. Also, these studies excluded from their subjects teeth with ⅔ or less formed roots. In the present study, ⅔ formed teeth ERR prevalence is 38,2%; less formed teeth ERR prevalence is 66,1%. In another study, researchers found ERR prevalence of 24,2% on periapical radiographs (21). Considering that CBCTs provide more detailed examination than conventional radiographs and can detect more ERR (6), it can be considered to be compatible with the percentages in our study. Even researchers showed histological resorption in all second molar teeth adjacent to the impacted third molar (22); the rate of 54.3% remains reasonable.

According to some previous studies, it is suggested that more ERR is seen in men than women, possibly due to hormonal differences (23,24). However, in our study, gender proved not to be a predictive risk factor for ERR.

In our study, impacted third molars with horizontal and mesioangular position, osseous retention, and Pell and Gregory Classes B and C, were more likely to cause root resorption in adjacent second molars. These results are similar to the previous studies (2,6,19,21,25). The lack of space for the third molars to erupt is cited as the main reason for the third molars to remain impacted (26). As a result of this narrowness of space, the authors believe that the pressure of the impacted third molar, which is trying to erupt into the second molar, causes resorption, especially in mesioangular and horizontal situations. Previous research stated that the eruptive movements do not stop after root formation is completed. These impacted third molars continue to put mechanical pressure on adjacent second molars, thus stimulating ERR formation and progression (19). Consistent with this view, no significant result was found between root formation degrees and ERR in our study. Results showed that incompletely formed roots also create ERR or can cause ERR somehow in the adjacent tooth.

A previous study observed that the pressure on the periodontal ligament and roots of the second molars decreases when the adjacent third molars have partially erupted (21). Our study's results support decreasing pressure opinion as more ERR cases were seen in osseous retention cases. At the same time, researchers indicate more ERR in Class B and C cases. In contrast, Oenning *et al*. found that ERR was seen more in Pell and Gregory Class A and Class B cases (4). These findings highlight that the cervical third of the tooth, namely the cementoenamel junction, is more prone to the inflammatory process resulting in ERR (23). In our research, the middle or apical third shows statistically significant ERR prevalence.

Our study found no significant difference between mesioangular and horizontal positions. This result agreed with the conclusion that a slightly larger contact surface between teeth in horizontal impaction may not represent an essential component for ERR formation in second molars (4). When ERR is detected, extracting the third molar can protect the second molar. In cases of severe ERR, if the third molar eruption seems possible, extracting the second molar may be another treatment method (15).

Previous studies, which examine periapical radiographs (23), panoramic radiographs (5), and CBCT (6), revealed higher ERR risk in mandibular second molars. In contrast to the aforementioned analyses, in our study, no statistical difference was found in the ratios of the maxilla and mandible.

Wang *et al*. stated that being over the age of 35 is an independent risk factor for ERR (19). ERR is a progressive condition because movement continues throughout life with mesialization and third molar eruption, and mechanical pressure on the second molar stimulates ERR progression (19,27). However, our study mainly examined early ages, so we could not consider aging a significant risk factor.

A limitation of our study was the accurate separation of ERR from dental caries. The studies investigating the contact area have mentioned this limitation (6). The presence of a space between the second and third molars that may cause food retention, especially in the case of mucosal retention, is an environment promoting dental caries (3,6). In a cross-sectional study, researchers indicate that there is contact between the second and third molars in cases of ERR in the cervical third, and there is no contact in cases of caries (28). In our study, we examined the retention status of the impacted tooth and the tooth-tooth contact. There was no resorption or caries in non-contact cases. Therefore, there is no disorder in the diagnosis. Another limitation of our study is that there is only a radiographic examination of the ERR. Therefore, to produce better scientific results, researchers should perform clinical observations with histopathological evaluation after tooth extraction and radiographic examination. Lastly, although the observers are experienced in radiological examinations in such narrow areas, optical illusions might occur. The proximity of the enamel of the impacted third molar to the examined area stands out as one of the effective factors for misdiagnosing.

Future research with a more heterogeneous population selection would be more appropriate to obtain a consensus on risk factors. In addition, investigating which conditions provoke ERR progress or stop and whether the resorbed areas are repaired and remineralized after the tooth extraction would make an important contribution to the literature.

## Conclusions

Based on the limitations of the present study, the ERR risk was higher in mesioangular and horizontal inclination, Pell and Gregory Classes B and C, osseous retention, and a middle and cervical contact area. Therefore, a more careful examination should be done in these cases, with or without symptoms.
